# Medical and Philosophical Intelligence

**Published:** 1811-10

**Authors:** 


### MEDICAL AND PHILOSOPHICAL INTELLIGENCE.

We have been favoured by Mr. Wilson, No. 39, Strand, with a specimen of the nut of the Sassafras tree, of which a quantity has recently been imported from South America. It contains a considerable portion of the essential oil of Sassafras; is highly fragrant; and is strongly recommended as a grateful nutritious article of diet; it may be taken in substance, or in the more agreeable form of chocolate. It is said to be very beneficial in restoring exhausted strength; in cases of impaired appetite; asthma; and some cutaneous affections. In a future number we shall hope to detail further particulars of this article.

---

The Editors have received from Mr. Want the following extract of a letter from Mr. Mitchell, of Weddon, Somersetshire, formerly an eminent practitioner in that neighbourhood.

"I have read your paper on gout, published in the Med. and Phys. Journal for September last, with much pleasure, and hope it will have a tendency to prevent our implicitly treading in the steps of our ancestors, however great their authority. I have long been a martyr to rheumatic gout, and for *several months past* had an attack of it on one side of the face, wihch commenced regularly every day at two o'clock, and continued about twelve hours. I took bark, arsenic, guaiacum, &c. &c. without producing the least good effect. One night I took some calomel and opium, which had no effect on the bowels as usual, but eating a plate of currants on the following morning, I was seized with *a diarrhœa*, which lasted many hours, but it completely carried off my complaint, and I have since had no return of it."

---

Prize

Prize proposed by *la société de médecine de Bruxelles*, for 1812. At the meeting on the 6th of May 1811, it was determined that a gold medal, value 200 francs, shall be adjudged to the author of the best memoir on the following questions.

1. " Quelle est la nature et la cause de la maladie connue sous le nom de fièvre jaune ?

2. " Quels sont les symptomes qui caractérisent essentiellement cette fièvre ?

3. La jaunisse et le vomissement noir doivent ils être regardés comme des symptômes essentiels ou caractéristiques de cette maladie, ou seulement comme des symptômes accidentels ?

4. " Cette fièvre est-elle contagieuse ?

5. " Quels sont les moyens de s'en garantir ?

6. " Quels sont les moyens curatifs les plus efficaces ?"

The essays are to be written in Latin or French, addressed, post-paid, to J. J. Caroly, M. D. Secretary to the Society, before the first of May 1812. Each memoir must bear a motto, and b accompanied by a sealed note, containing the same motto, and the name of the author.—Esprit des Journeaux.

The following formula is given in l'Esprit des Journeaux, for March 1811, as an excellent febrifuge. The substances which compose it are well known, but their combination has never been published.—R. Mousse de corse mondée ℥ij. Barbotine (*semen santonicum*) ℥j. Sel d'Absinthe (potass. carbonatée) ℥ss. Eau Commune ℔i. Boil them for an instant, let the decoction stand till cold, strain and sweeten it with simple syrup, or syrup of lemon. The dose of this for children is two or three table-spoonfuls, repeated several times in the day, or during three mornings fasting; in this case the portion is divided into three parts, and one is taken each morning successively. The decoction or infusion may be made with only ℥viij of water, and as much lait doux may afterwards be added.

The French Institute has proposed for the subject of a prize, a determination of the specific heat of gasses.

---

In Augsburg and its vicinity, which are celebrated for good beer, it is customary to put into each cask a small bag of the herb Bennet, (Geum urbanum.)

---

*Dr. Thornton* is disposing of his valuable collections on Botany, consisting of the whole impression, with the plates, drawings, and letter-press, of the illustrations of Linnæus, the collection of portraits of celebrated Botanists, the copy-rights, &c. &c. of his various publications on the same subj ct, by way of Lottery. The excellence of many of the portraits as paintings, independent of the subject; the taste displayed in the botanical drawings, most of them by Reinagle; the immense sum expended in making the collection, and on its publication, certainly entitle Dr. Thornton to a patronage in which, we hope, on this occasion, he will find an ample remuneration.

Theatre of Anatomy.—Lectures on Anatomy, Physiology, Pathology, and Surgery, by Mr. John Taunton, F. A. S. Member of the Royal College of Surgeons of London, Surgeon to the City and Finsbury Dispensaries, City of London Truss Society, &c.—In this course of Lectures it is proposed to take a comprehensive view of the structure and economy of the living body, and to consider the causes, symptoms, nature, and treatment of surgical diseases, with the mode of performing the different surgical operations; forming a complete course of anatomical and physiological instruction for the medical and surgical student, the artist, the professional or private gentleman.

An ample field for professional edification will be afforded by the opportunity which pupils may have of attending the clinical and other practice of both the City and Finsbury Dispensaries.

The Winter course will commence on Saturday, October the 5th, 1811, at Eight o'clock in the evening precisely, and be continued every Tuesday, Thursday, and Saturday, at the same hour.

Particulars may be had, on applying to Mr. Taunton, Greville Street, Hatton Garden.

---

Lectures on Surgery, and on Physiology.—Mr. A. Carlisle, F.R.S. F.L.S. Professor of Anatomy in the Royal Academy, and Surgeon to the Westminster Hospital, will begin his course of Lectures on the Art and Practice of Surgery, on Tuesday, October the 8th, at eight o'clock in the evening, at his house in Soho Square. The subject will be continued on Tuesdays, Thursdays, and Saturdays, at the same hour.

The diseases and accidents allotted to the province of Surgery will be fully treated of and illustrated by cases from the lecturer's experience. The different operations will be demonstrated, and the anatomy of the parts explained.

These Lectures combine views of the Natural History, Physiology, and Pathology of the Human Body, calculated to illustrate the several processes of healing, and to afford a compendious view of the animal economy. The introductory discourse will be open to all students.

---

*London Infirmary for curing Diseases of the Eye.*—J. R. Farre, Physician.—B. Travers, Surgeon. The practice of this Institution is open to Students. The terms of attendance may be known by application to Mr. Murley, 79, St. Paul's Church-yard.

---

Dr. R. Watt, of Glasgow, will begin his Winter course of Lectures on the Practice of Medicine, on Wednesday, the 6th Nov. at nine in the morning. On the same day, at ten in the morning, he will begin a separate course on the Theory of Medicine. Each course will continue five days a week for six months.

---

Mr. Allan Burns, of Glasgow, will commence his course of Lectures tures on Anatomy, and on the Principles and Operations of Surgery, on Tuesday, the 5th of November, at five o'clock in the afternoon.

---

Dr. Paris will recommence his course of Lectures on Pharmaceutic Chemistry, on Tuesday, the 8th of October, at the Westminster Hospital, at eight o'clock in the evening. The plan of the course may be learnt by applying to Mr. Callow, or to Mr. Merrick, Apothecary of the Westminster Hospital, from whom a syllabus of the course may be obtained.

---

Dr. Merriman's course of Lectures on the Theory and Practice of Midwifery, and the Diseases of Women and Children, will commence on Monday, October 7, at his house in Curzon Street, Mayfair.

---

Dr. Squire will, on Saturday, the 5th October, begin a course of Lectures on the Theory and Practice of Midwifery, and the Diseases of Women and Children.

Particulars may be known by applying to Dr. Squire, 30, Ely-Place, Holborn.

---

Early in November, Mr. Allan Burns, of Glasgow, will publish "Observations on the Surgical Anatomy of the Head and Neck, illustrated by numerous cases and engravings." This work will be composed in one volume, 8vo.

---

In the Press.—Speculations, &c. on the effects of counter Irritation in a variety of Diseases of the Human Frame. By M. Brown, of Banbury.

Practical Remarks on Insanity; by Bryan Crowther, Surgeon to Bethlem Hospital.

In the Press, and shortly will be published, by James Gillman, Surgeon, Highgate, "An Essay on the Bite of a Rabid Animal;" being the Substance of an Essay which received a prize from the Royal College of Surgeons.

